# Changes in overweight/obesity and central obesity status from preadolescence to adolescence: a longitudinal study among schoolchildren in Japan

**DOI:** 10.1186/s12889-020-8343-3

**Published:** 2020-02-17

**Authors:** Hirotaka Ochiai, Takako Shirasawa, Rimei Nishimura, Takahiko Yoshimoto, Akira Minoura, Kosuke Oikawa, Ayako Miki, Hiromi Hoshino, Akatsuki Kokaze

**Affiliations:** 1 Department of Hygiene, Public Health and Preventive Medicine, Showa University School of Medicine, 1-5-8 Hatanodai, Shinagawa-ku, Tokyo, 142-8555 Japan; 2 Division of Diabetes, Metabolism and Endocrinology, Department of Internal Medicine, Jikei University School of Medicine, 3-25-8 Nishi-Shinbashi Minato-ku, Tokyo, 105-8461 Japan

**Keywords:** Overweight/obesity, Central obesity, Preadolescence, Adolescence, Longitudinal study

## Abstract

**Background:**

Little is known about changes in overweight/obesity and central obesity status among schoolchildren from preadolescence to adolescence in Japan, where waist circumference (WC) is generally not measured in annual health examinations at elementary and junior high schools. This study examined changes of overweight/obesity and central obesity status among schoolboys and schoolgirls from preadolescence to adolescence in Japan.

**Methods:**

Study subjects were fourth-grade school children (9 or 10 years of age) from all four of Ina town’s elementary schools in Japan. Measurement of each participant’s height, weight, and WC were made at baseline and 3 years later. Childhood overweight/obesity was determined according to the age- and sex-specific body mass index cut-off points proposed by the International Obesity Task Force. Central obesity was defined as waist-to-height ratio ≥ 0.5. Kappa (κ) statistic was calculated to examine the tracking of overweight/obesity and central obesity.

**Results:**

Data from 1436 participants (boys: *n* = 720, girls: *n* = 716) were analyzed. Overweight/obesity status tracked substantially from fourth grade to seventh grade in both boys (κ = 0.614, *P* value < 0.001) and girls (κ = 0.619, *P* value < 0.001). Among participants who were overweight/obese in fourth grade, 55.2% of boys and 63.2% of girls were still overweight/obese in seventh grade. Tracking of central obesity from fourth graders to seventh graders was substantial in boys (κ = 0.651, *P* value < 0.001) and moderate in girls (κ = 0.544, *P* value < 0.001). Among participants who had central obesity in fourth grade, 54.1% of boys and 52.6% of girls still had central obesity in seventh grade.

**Conclusions:**

The present study showed that the tracking of overweight/obesity from preadolescence to adolescence was substantial in boys and girls. Moreover, more than half of those who had central obesity in preadolescence had central obesity in adolescence. This study suggests that it is important to implement a primary prevention program for overweight/obesity and central obesity in elementary schools before fourth grade.

## Introduction

Childhood overweight/obesity is an important public health problem because comorbidities such as elevated blood pressure, dyslipidemia, and type 2 diabetes occur frequently in the overweight and obese pediatric population [1]. In addition, a previous study in Japan showed that about 32% of obese boys and 41% of obese girls became obese adults [2]. Moreover, overweight and obesity in childhood and adolescence have adverse consequences on premature mortality and physical morbidity in adulthood [3]. Therefore, it is necessary to prevent childhood overweight/obesity for current and future health.

Central obesity is a strong risk factor for metabolic disorders and cardiometabolic diseases in children and adolescents [4]. In addition, a recent study showed that central obesity is a better predictor of the presence of cardiovascular risk factors than obesity [5]. Furthermore, a previous study showed that central obesity was associated with higher rates of school absence, more visits to a physician, and lower health-related quality of life in children [6]. Thus, it is important to prevent central obesity in addition to overweight/obesity in children and adolescents.

It is important to consider the changes of overweight/obesity and central obesity in children over time in order to design an effective prevention program. To date, several studies have reported on these changes. Leitão et al. showed trajectories of obesity from late childhood to adolescence [7]. Chrzanowska et al. reported the tracking of abdominal obesity from the age of 7 to 15 years [8]. However, little is known about changes of both overweight/obesity and central obesity status during the period from preadolescence to adolescence in Japan, where waist circumference (WC) is not commonly measured in annual health examinations at elementary and junior high schools.

The aim of this study was to examine changes of overweight/obesity status and central obesity status among schoolboys and schoolgirls from preadolescence to adolescence in Japan.

## Methods

### Subjects

Study subjects were 1607 fourth-grade school children (9 or 10 years of age) from all four of Ina town’s elementary schools between 2004 and 2007. Ina-town, in Saitama Prefecture, Japan, had implemented a unique health check-up program as a part of its community health services. The present study was conducted as a part of that program. Anthropometric measurements were performed as part of that program. Details of these measurements are described below. Written informed consent was obtained from the parent or guardian of each subject prior to the subject’s participation in this study. This study protocol was approved by the Medical Ethics Committee of Showa University School of Medicine (Approval No. 127).

Among 1607 subjects, 8 refused to participate in the program (participation rate: 99.5%). Of 1599 children, 1441 underwent follow-up measurements at seventh grade (follow-up rate: 90.1%), and 5 were excluded due to missing data regarding variables in this study. Thus, data from 1436 participants (boys: *n* = 720, girls: *n* = 716) were analyzed.

### Anthropometric measurements

Measurements of each participant’s height, weight, and WC were conducted by trained staff either in the school’s infirmary or in a designated room to protect privacy. Participants wore light clothing but no shoes or socks. Height was measured to the nearest 0.1 cm using a stadiometer (TK-11253, Takei Scientific Instruments Co., Ltd., Niigata, Japan), and weight was measured to the nearest 0.1 kg using a scale (Model TBF-102, Tanita, Tokyo, Japan). Body mass index (BMI) was calculated as weight (kg) divided by height (m) squared. In the measurement of WC, participants were asked to hold the arms in a relaxed position at the sides and breathe normally for a few breaths [9]. Then, the measurement was taken at the end of a normal expiration. WC was measured to the nearest 0.1 cm using a measuring tape (070117, Artec Co., Ltd., Osaka, Japan) in a standing position at the navel level while another examiner checked verticality from the side. The measurements of height, weight, and WC were performed one time for each participant. Waist-to-height ratio (WHtR) was calculated as WC (cm)/height (cm). All measurements were conducted using the same procedures at fourth and seventh grade.

### Definition of overweight/obesity and central obesity

Childhood overweight and obesity were determined according to the age- and sex-specific BMI cut-off points proposed by the International Obesity Task Force (IOTF) [10]. The cut-off points, which was based on the data including Asian people, have been used in many epidemiological studies for Japanese children and adolescents [11–15]. In the present study, overweight and obesity were combined into the single variable overweight/obesity because the number of participants with obesity was small. In accordance with previous studies [4, 16], central obesity was defined as WHtR ≥0.5.

### Data analysis

Statistical analysis was performed separately for each sex. Data are presented as median (25 percentile, 75 percentile) for continuous variables or number (%) for categorical variables. Wilcoxon’s rank-sum test or chi-square test was used to compare characteristics between boys and girls.

Based on previous studies [7, 17, 18], the kappa (κ) statistic was calculated to examine the tracking of overweight/obesity and central obesity. For instance, if participants who had central obesity in fourth grade still had central obesity in seventh grade, it was considered as tracking of central obesity. According to previous studies [18–20], the tracking was interpreted as follows; slight (0 ≤ κ ≤ 0.20), fair (0.21 ≤ κ ≤ 0.40), moderate (0.41 ≤ κ ≤ 0.60), substantial (0.61 ≤ κ ≤ 0.80), and almost perfect (0.81 ≤ κ ≤ 1).

A *P* value < 0.05 was considered statistically significant. All statistical analyses were performed using Statistical Analysis System (SAS) software (version 9.4; SAS Institute Inc., Cary, NC, USA).

## Results

Characteristics of study participants in fourth and seventh grade are shown in Table 1. In fourth grade, BMI, WC, and WHtR were significantly higher in boys than in girls. In seventh grade, BMI and WHtR were significantly higher in girls than in boys. There were statistically significant differences between boys and girls in the proportion of overweight/obesity and central obesity in fourth grade. In contrast, no significant differences between boys and girls were observed in the proportion of overweight /obesity and central obesity in seventh grade.

**Table 1:** Characteristics of study participants at baseline (fourth grade) and 3 years later (seventh grade)

	In fourth grade			In seventh grade		
	Boys (*n* = 720)	Girls (*n* = 716)	*P* value^a^	Boys (*n* = 720)	Girls (*n* = 716)	*P* value^a^
Age (years)	9.0 (9.0, 10.0)	9.0 (9.0, 10.0)	0.764	12.0 (12.0, 12.0)	12.0 (12.0, 12.0)	0.142
Height (cm)	134.9 (131.1, 139.0)	134.4 (130.6, 139.5)	0.397	153.6 (147.7, 158.8)	152.2 (148.5, 156.0)	< 0.001
Weight (kg)	30.3 (27.4, 34.9)	29.5 (26.3, 34.0)	0.001	42.5 (37.7, 48.5)	42.6 (38.3, 47.4)	1.000
Body mass index (kg/m^2^)	16.7 (15.5, 18.5)	16.2 (15.2, 17.8)	< 0.001	17.9 (16.7, 19.4)	18.2 (17.0, 19.9)	0.010
Waist circumference (cm)	57.7 (54.4, 62.7)	57.3 (54.3, 61.0)	0.031	63.0 (59.6, 67.3)	63.7 (60.0, 67.7)	0.073
Waist-to-height ratio	0.428 (0.407, 0.459)	0.425 (0.407, 0.452)	0.038	0.41 (0.39, 0.43)	0.42 (0.40, 0.44)	< 0.001
Overweight/obesity	125 (17.4)	87 (12.2)	0.005	82 (11.4)	79 (11.0)	0.831
Central obesity	85 (11.8)	57 (8.0)	0.015	50 (6.9)	47 (6.6)	0.774

**Except where indicated n (%), values are median (25th percentile, 75th percentile)**

^**a**^**Wilcoxon’s rank-sum test or chi-square test**

Table 2 shows comparisons between boys and girls in the proportion of participants with overweight/obesity in seventh grade. No statistically significant differences were seen between groups. Results were similar even if the analysis was limited to non-overweight/obesity participants in fourth grade or overweight/obesity participants in fourth grade. In those with overweight/obesity in fourth grade, 55.2% of boys and 63.2% of girls still had overweight/obesity in seventh grade.

**Table 2:** The proportion with overweight/obesity in seventh grade by sex

	Boys	Girls	*P* value^a^
Total participants in fourth grade	*n* = 720	*n* = 716	
Non-overweight/obesity in seventh grade	638 (88.6)	637 (89.0)	0.831
Overweight/obesity in seventh grade	82 (11.4)	79 (11.0)	
Non-overweight/obesity participants in fourth grade	*n* = 595	*n* = 629	
Non-overweight/obesity in seventh grade	582 (97.8)	605 (96.2)	0.096
Overweight/obesity in seventh grade	13 (2.2)	24 (3.8)	
Overweight/obesity participants in fourth grade	*n* = 125	*n* = 87	
Non-overweight/obesity in seventh grade	56 (44.8)	32 (36.8)	0.244
Overweight/obesity in seventh grade	69 (55.2)	55 (63.2)	

^**a**^**Chi-square test**

Tracking of overweight/obesity from fourth graders to seventh graders was demonstrated by sex (Table 3). The proportion of boys with overweight/obesity in both fourth and seventh grade was 9.6%, whereas the proportion without overweight/obesity in both fourth and seventh grade was 80.8%. In girls, the proportion with overweight/obesity in both fourth and seventh grade was 7.7%, whereas the proportion without overweight/obesity in both fourth and seventh grade was 84.5%. The κ statistics were 0.614 in boys and 0.619 in girls. When obesity was determined by the percentage of overweight [21], which is used in national statistics of school health in Japan, the κ statistics were 0.575 in boys and 0.520 in girls.

**Table 3:** Tracking of overweight/obesity from fourth to seventh grade by sex

	Overweight/obesity in seventh grade		κ	*P* value
	Yes	No		
Boys (*n* = 720)				
Overweight/obesity in fourth grade				
Yes	69 (9.6)	56 (7.8)	0.614	< 0.001
No	13 (1.8)	582 (80.8)		
Girls (*n* = 716)				
Overweight/obesity in fourth grade				
Yes	55 (7.7)	32 (4.5)	0.619	< 0.001
No	24 (3.4)	605 (84.5)		

**κ: Kappa**

The proportion with central obesity in seventh grade was also compared between boys and girls (Table 4). No statistically significant differences were seen. A statistically significant difference was observed between boys and girls who had non-central obesity in fourth grade in the proportion with central obesity in seventh grade (0.6% vs. 2.6%, respectively; *P* = 0.006). In contrast, no statistically significant differences were observed between boys and girls with central obesity in fourth grade in the proportion with central obesity in seventh grade. Among those with central obesity in fourth grade, 54.1% of boys and 52.6% of girls still had central obesity in seventh grade.

**Table 4:** The proportion with central obesity in seventh grade by sex

	Boys	Girls	*P* value^a^
Total participants in fourth grade	*n* = 720	*n* = 716	
Non-central obesity in seventh grade	670 (93.1)	669 (93.4)	0.774
Central obesity in seventh grade	50 (6.9)	47 (6.6)	
Non-central obesity participants in fourth grade	*n* = 635	*n* = 659	
Non-central obesity in seventh grade	631 (99.4)	642 (97.4)	0.006
Central obesity in seventh grade	4 (0.6)	17 (2.6)	
Central obesity participants in fourth grade	*n* = 85	*n* = 57	
Non-central obesity in seventh grade	39 (45.9)	27 (47.4)	0.862
Central obesity in seventh grade	46 (54.1)	30 (52.6)	

^**a**^**Chi-square test**

Table 5 shows tracking of central obesity from fourth graders to seventh graders. Among boys, the proportion with non-central obesity in fourth and seventh grade was 87.6%, whereas the proportion with central obesity in fourth and seventh grade was 6.4%. The κ statistic was 0.651. The proportion of girls with non-central obesity in fourth and seventh grade was 89.7%, whereas the proportion with central obesity in fourth and seventh grade was 4.2%. The κ statistic was 0.544.

**Table 5:** Tracking of central obesity from fourth to seventh grade by sex

	Central obesity in seventh grade		κ	*P* value
	Yes	No		
Boys (*n* = 720)				
Central obesity in fourth grade				
Yes	46 (6.4)	39 (5.4)	0.651	< 0.001
No	4 (0.6)	631 (87.6)		
Girls (*n* = 716)				
Central obesity in fourth grade				
Yes	30 (4.2)	27 (3.8)	0.544	< 0.001
No	17 (2.4)	642 (89.7)		

**κ: Kappa**

In a logistic regression analysis, the odds ratios of overweight/obesity at fourth grade for being overweight/obesity at seventh grade were 55.16 among boys and 43.33 among girls, which were statistically significant. The odds ratios of central obesity at fourth grade for being central obesity at seventh grade were 186.06 among boys and 41.95 among girls, which were statistically significant.

## Discussion

The present study investigated changes in overweight/obesity status and central obesity status from preadolescence to adolescence among schoolchildren in Japan. Results showed that boys and girls had a similar tendency in terms of the development of overweight/obesity. In contrast, there was a statistically significant difference between boys and girls in terms of the development of central obesity. To the best of our knowledge, this is the first study regarding changes of overweight/obesity and central obesity status from fourth to seventh grade among Japanese schoolchildren. However, the present study findings should be carefully discussed.

In this study, the prevalence of overweight/obesity in fourth grade (median age: 9.0 years) was 17.4% among boys and 12.2% among girls. Rangelova et al. showed that the prevalence of overweight/obesity was 30.4% in boys and 28.3% in girls among Bulgarian schoolchildren aged 6–9 years [22]. Khadilkar et al. reported that the prevalence of overweight/obesity in boys was 19.6% and that in girls was 18.3% among Indian children aged 6–9 years [23]. Kelishadi et al. showed that the prevalence of overweight/obesity was 7.1% for boys and 8.6% for girls in Iranian children aged 9 years [24]. In these studies [22–24], overweight/obesity was defined by cut-off points established by the IOTF, which was same as for this study. Therefore, the prevalence of overweight/obesity in our study was different from that in studies conducted in the other countries, which could limit the ability to generalize our study findings to other countries.

The prevalence of central obesity (WHtR ≥0.5) in our study was 11.8% in boys and 8.0% in girls aged 9–10 years. Okuma et al. reported that the prevalence of central obesity among Japanese children (mean age: 10.9 years) was 16.5% in boys and 10.6% in girls [25]. A recent study showed that the prevalence of central obesity (WHtR ≥0.5) among Spanish children aged 6 to 11 years was 21.3% (24.6% for boys and 17.9% for girls) [26]. The prevalence of central obesity defined by WHtR ≥0.5 among Portuguese children (mean age: 9.3 years) was reported to be 28.1% for boys and 19.4% for girls [27]. Thus, the prevalence of central obesity among children in these studies [25–27] was higher than in our study. Central obesity has been reported to be associated with dietary habits and physical activity [4, 28]. In addition, Zhang et al. demonstrated that central obesity among children is associated with affluence and urban residence [29]. Because these factors might affect the difference in the prevalence of central obesity between our study and other studies, future studies are needed to elucidate the difference.

In our study, overweight/obesity status tracked substantially from fourth to seventh grade in both sexes. Moreover, more than half of participants who had overweight/obesity in fourth grade still had overweight/obesity in seventh grade in both sexes. In addition, overweight/obesity at fourth grade significantly increased the odds ratio for being overweight/obesity at seventh grade. These findings suggest that it is important to prevent overweight/obesity before fourth grade. Because schools are in a unique position to address the issue of childhood obesity [30], it is necessary to implement a primary prevention program for overweight/obesity in elementary schools.

Among participants with non-central obesity in fourth grade, a statistically significant difference between boys and girls was observed in the proportion with central obesity in seventh grade in our study; girls were more likely to develop central obesity than boys. These results suggest the presence of sex differences in the development of central obesity from preadolescence to adolescence. The sex difference might be due to the difference in growth between boys and girls because Wells reported that sex differences in body composition are primarily attributable to the action of sex steroid hormones, which drive the differences during pubertal development [31]. In addition, more than 50% of those with central obesity in fourth grade still had central obesity in seventh grade regardless of sex. Moreover, central obesity at fourth grade significantly increased the odds ratio for being central obesity at seventh grade. Thus, a primary prevention program for central obesity is needed before fourth grade, and it is necessary to focus more on girls than boys in a prevention program for central obesity during the period from preadolescence to adolescence.

The strength of this study was that height, weight, and WC were measured for more than 1000 schoolchildren, and these values were used to define overweight/obesity and central obesity. Moreover, the 3-year follow-up rate was more than 90%. However, there are a few limitations to this study. First, information regarding sex hormones, socio-economic status, environment, food intake, and KAPs (knowledge, attitude, and practice) was not obtained in this study. Therefore, the possibility of residual confounding cannot be ruled out. Second, height, weight, and WC were not measured by a single measurer. Thus, the error of measurement could influence the present study results. Finally, this study was based on a local data collected as a part of community health services in one town in Japan. Therefore, the data was not sampled as a representative for Japan, which might limit the generalizability to other populations.

## Conclusions

The present study showed that tracking of overweight/obesity was substantial in boys and girls from preadolescence to adolescence. Moreover, more than half of those with central obesity in preadolescence still had central obesity in adolescence. Accordingly, this study suggests that it is important to implement a primary prevention program for overweight/obesity and central obesity in elementary schools, especially before fourth grade. Future longitudinal studies will be needed to provide more evidence for changes of overweight/obesity status and central obesity status among schoolchildren from preadolescence to adolescence in Japan. It is necessary to address limitations of this study (potential confounders, the error of measurement, and the sampling selection) in the future researches.

## Data Availability

The datasets used and/or analysed during the current study are available from the corresponding author on reasonable request and only after approval from the Medical Ethics Committee of Showa University School of Medicine.

## Abbreviations

BMI: Body mass index
IOTF: International obesity task force
WC: Waist circumference
WHtR: Waist-to-height ratio
κ: Kappa

## References

[1] Deckelbaum RJ, Williams CL (2001). Childhood obesity: the health issue. Obes Res.

[2] Kotani K, Nishida M, Yamashita S, Funahashi T, Fujioka S, Tokunaga K (1997). Two decades of annual medical examinations in Japanese obese children: do obese children grow into obese adults?. Int J Obes Relat Metab Disord.

[3] Reilly JJ, Kelly J (2011). Long-term impact of overweight and obesity in childhood and adolescence on morbidity and premature mortality in adulthood: systematic review. Int J Obes.

[4] Grigorakis DA, Georgoulis M, Psarra G, Tambalis KD, Panagiotakos DB, Sidossis LS (2016). Prevalence and lifestyle determinants of central obesity in children. Eur J Nutr.

[5] Hassapidou M, Tzotzas T, Makri E, Pagkalos I, Kaklamanos I, Kapantais E (2017). Prevalence and geographic variation of abdominal obesity in 7- and 9-year-old children in Greece; World Health Organization childhood obesity surveillance initiative 2010. BMC Public Health.

[6] Kesztyus D, Wirt T, Kobel S, Schreiber A, Kettner S, Dreyhaupt J (2013). Is central obesity associated with poorer health and health-related quality of life in primary school children?. Cross-sectional Results Baden-Wurttemberg Study BMC Public Health.

[7] Leitao R, Rodrigues LP, Neves L, Carvalho GS (2011). Changes in adiposity status from childhood to adolescence: a 6-year longitudinal study in Portuguese boys and girls. Ann Hum Biol.

[8] Chrzanowska M, Suder A, Kruszelnicki P (2012). Tracking and risk of abdominal obesity in the adolescence period in children aged 7-15. The Cracow longitudinal growth study. Am J Hum Biol.

[9] World Health Organization. STEPS Manual. 2017. https://www.who.int/ncds/surveillance/steps/manual/en/.

[10] Cole TJ, Bellizzi MC, Flegal KM, Dietz WH (2000). Establishing a standard definition for child overweight and obesity worldwide: international survey. BMJ..

[11] Ikeda N, Fuse K, Nishi N (2017). Changes in the effects of living with no siblings or living with grandparents on overweight and obesity in children: results from a national cohort study in Japan. PLoS One.

[12] Kachi Y, Otsuka T, Kawada T (2015). Socioeconomic status and overweight: a population-based cross-sectional study of Japanese children and adolescents. J Epidemiol..

[13] Liu J, Sekine M, Tatsuse T, Hamanishi S, Fujimura Y, Zheng X (2014). Family history of hypertension and the risk of overweight in Japanese children: results from the Toyama birth cohort study. J Epidemiol.

[14] Murakami K, Miyake Y, Sasaki S, Tanaka K, Arakawa M (2011). Dietary glycemic index and glycemic load in relation to risk of overweight in Japanese children and adolescents: the Ryukyus child health study. Int J Obes.

[15] Murakami K, Miyake Y, Sasaki S, Tanaka K, Arakawa M (2012). Self-reported rate of eating and risk of overweight in Japanese children: Ryukyus Child Health Study. J Nutr Sci Vitaminol (Tokyo).

[16] Mokha JS, Srinivasan SR, Dasmahapatra P, Fernandez C, Chen W, Xu J (2010). Utility of waist-to-height ratio in assessing the status of central obesity and related cardiometabolic risk profile among normal weight and overweight/obese children: the Bogalusa heart study. BMC Pediatr.

[17] Deshmukh-Taskar P, Nicklas TA, Morales M, Yang SJ, Zakeri I, Berenson GS (2006). Tracking of overweight status from childhood to young adulthood: the Bogalusa heart study. Eur J Clin Nutr.

[18] Kristiansen AL, Bjelland M, Brantsaeter AL, Haugen M, Meltzer HM, Nystad W (2015). Tracking of body size from birth to 7 years of age and factors associated with maintenance of a high body size from birth to 7 years of age--the Norwegian mother and child cohort study (MoBa). Public Health Nutr.

[19] Lamarre AK, Rascovsky K, Bostrom A, Toofanian P, Wilkins S, Sha SJ (2013). Interrater reliability of the new criteria for behavioral variant frontotemporal dementia. Neurology..

[20] Landis JR, Koch GG (1977). The measurement of observer agreement for categorical data. Biometrics..

[21] Dobashi K (2016). Evaluation of obesity in school-age children. J Atheroscler Thromb.

[22] Rangelova L, Petrova S, Konstantinova M, Duleva V, Dimitrov P (2014). Overweight and obesity prevalence in Bulgarian schoolchildren: a comparison between two international standards. Int J Biomed Adv Res.

[23] Khadilkar VV, Khadilkar AV, Cole TJ, Chiplonkar SA, Pandit D (2011). Overweight and obesity prevalence and body mass index trends in Indian children. Int J Pediatr Obes.

[24] Kelishadi R, Ardalan G, Gheiratmand R, Majdzadeh R, Hosseini M, Gouya MM (2008). Thinness, overweight and obesity in a national sample of Iranian children and adolescents: CASPIAN study. Child Care Health Dev.

[25] Okuma H, Okada T, Abe Y, Saito E, Iwata F, Hara M (2013). Abdominal adiposity is associated with high-density lipoprotein subclasses in Japanese schoolchildren. Clin Chim Acta.

[26] Schroder H, Ribas L, Koebnick C, Funtikova A, Gomez SF, Fito M (2014). Prevalence of abdominal obesity in Spanish children and adolescents. Do we need waist circumference measurements in pediatric practice?. PLoS One.

[27] Albuquerque D, Nobrega C, Samouda H, Manco L (2012). Assessment of obesity and abdominal obesity among Portuguese children. Acta Medica Port.

[28] Suder A, Janusz M, Jagielski P, Glodzik J, Palka T, Cison T (2015). Prevalence and risk factors of abdominal obesity in polish rural children. Homo..

[29] Zhang YX, Zhao JS, Chu ZH, Tan HL (2014). Prevalence and regional disparities in abdominal obesity among children and adolescents in Shandong, China, surveyed in 2010. Ann Nutr Metab.

[30] Probart C, McDonnell E, Weirich JE, Birkenshaw P, Fekete V (2007). Addressing childhood overweight through schools. Coll Antropol.

[31] Wells JC (2007). Sexual dimorphism of body composition. Best Pract Res Clin Endocrinol Metab.

